# Craniofacial fibrous dysplasia associated with McCune-Albright syndrome: challenges in diagnosis and treatment: case reports

**DOI:** 10.1186/s12903-019-0872-8

**Published:** 2019-08-08

**Authors:** Theodora Miti Kabali, Jeremiah Robert Moshy, Sira Stanslaus Owibingire, Karpal Singh Sohal, Elison N. M. Simon

**Affiliations:** 1 Dental department, Litete District Hospital, Kabwe, Zambia; 2 Department of oral and maxillofacial surgery, Muhimbili University of Health and Allied Sciences, P.O. Box 65001, Dar es Salaam, Tanzania

**Keywords:** Craniofacial fibrous dysplasia, McCune-Albright syndrome, Morbidity, Case report

## Abstract

**Background:**

McCune-Albright syndrome (MAS) is a rare multisystem disorder that classically was defined by the triad of polyostotic fibrous dysplasia of bone, café-au-lait skin pigmentation, and precocious puberty. It is a condition that has a gradual onset, slow growth rate and remain painless throughout. The clinical phenotype of MAS is highly variable and no definite treatment is available.

**Case presentation:**

This article describes two cases, a 10-year-old girl and an 11-year-old boy, both with MAS comprising deforming craniofacial FD. Challenges related to diagnosis and management included late reporting with big lesions, involvement of multiple craniofacial bones, mutilating surgeries and ultimately high degree of morbidity.

**Conclusion:**

Delayed diagnosis and management of MAS results in devastating physical disabilities and severe morbidity after treatment.

## Background

Craniofacial fibrous dysplasia (FD) may present with varying severity ranging from being asymptomatic to causing severe disfigurement and impairment of function [1]. It is often severe in McCune-Albright syndrome (MAS) patients [2].

McCune-Albright syndrome was initially described separately by Donovan McCune and Fuller Albright in children [3, 4]. It is considered to be a relatively rare multisystem disorder with an estimated prevalence between 1/100,000 and 1/1,000,000, and is more common in females. Formerly, it was characterized by the presence of polyostotic fibrous dysplasia of bone (FD), café-au-lait skin pigmentation, and precocious puberty [5]. However, It was later recognized that other endocrinopathies including hyperthyroidism, excessive growth hormone (GH), renal phosphate loss with or without rickets/osteomalacia and Cushing’s syndrome could be found in association with the basic features [6].

The disorder is the result of inherent activation of adenylyl cyclase and overproduction of 3′,5′-cyclic adenosine monophosphate (cAMP) which is attributed to post-zygotic somatic mutation in the gene GNAS 1 on chromosome 20q13–13.29 that codes for the alpha subunit of stimulatory G protein [4, 7]. It is hypothesized that this disorder is secondary to postzygotic mutations and that patients are somatic mosaics supported by the lack of vertical transmission of the disease and skin and bone lesions that rarely cross the midline [6].

The clinical phenotype of MAS is highly variable, depending upon the location and timing of the mutation during embryologic development [8], and the number of mutated cells and affected organs [4]. In MAS, among the most obvious signs is presence of skin lesions which are irregular, pigmented single or multiple flat macules on the skin referred to as the café-au-lait spots. These tan-brown hyper pigmented spots develop during infancy and become more obvious with age or sun exposure [3]. Precocious puberty is more common in girls than boys and is caused by spontaneous development of functioning ovarian cysts. Clinically, precocious puberty presents with vaginal bleeding, early breast development, and growth acceleration [7].

**Fig. 1:**
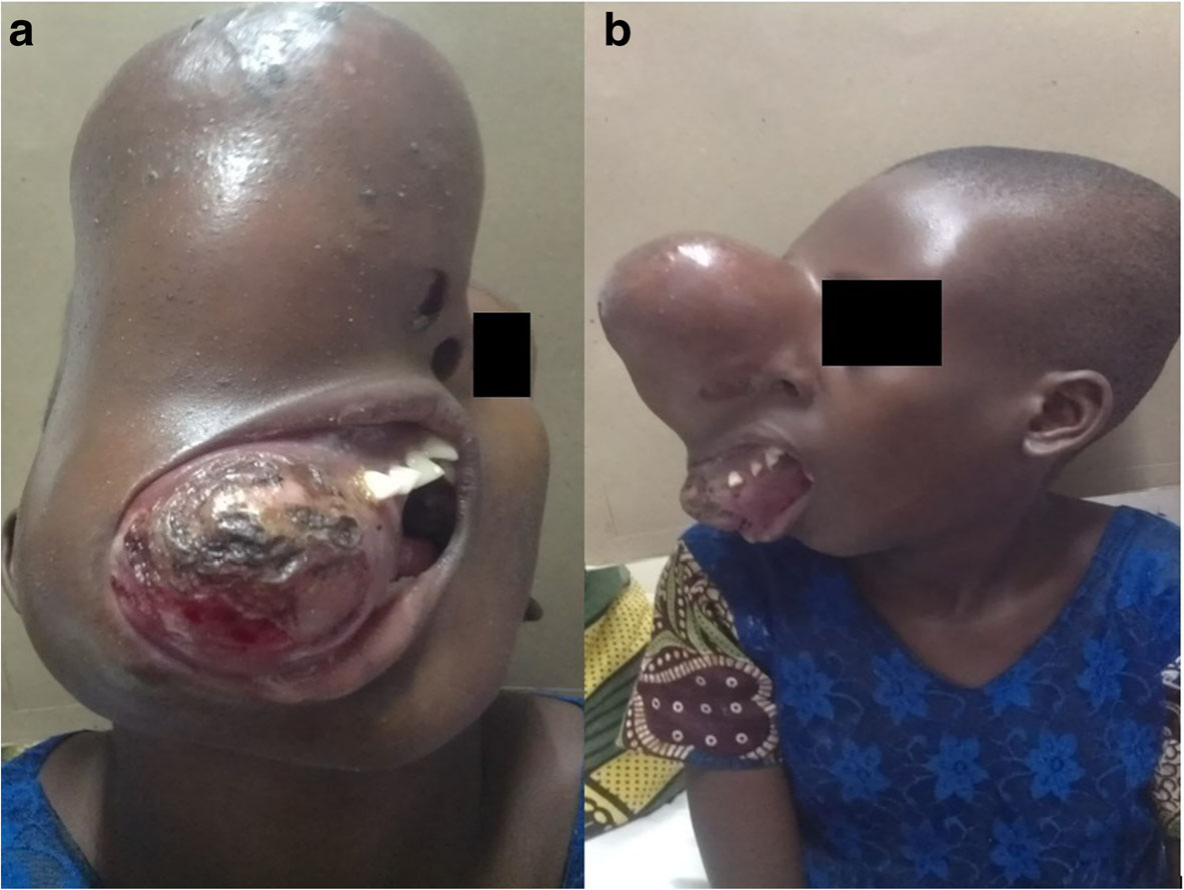
**a** & **b**: Case 1 showing extensive craniofacial lesion

Although prenatal diagnosis is not possible and no definite treatment is available for MAS, recently, through novel polymerase chain reaction-based techniques, activating mutation in the peripheral blood of patients with MAS has been successfully detected [4].This might help in diagnostic as well therapeutic areas. Nevertheless, diagnosis of MAS in developing countries is further challenging because of several factors including late reporting of the patients to the health facilities (Fig. 1 and Fig. 4), lack of sophisticated diagnostic facilities, and high cost of investigations. Diagnosis is often reached with the aid of different imaging techniques including plain x-rays, bone scintigraphy and magnetic resonance imaging [9].

We describe two cases of MAS seen at the oral and maxillofacial surgery unit of the Muhimbili National Hospital in Dar-es-Salaam, Tanzania between 2015 and 2018. They both presented with a heavy disease burden comprising deforming craniofacial and axial skeletal FD, precocious puberty and café-au-lait spots that contributed to poor quality of life. We describe the challenges in diagnosis and management of such cases in a developing country setting.

## Case reports

### Case 1

A 10-year-old female patient presented with complaint of a painless facial swelling for about 5 years. According to her parent, the swelling started as a small roundish and hard mass around the area below the right eye. It gradually, but consistently increased in size resulting in severe facial deformity. There was no significant medical history apart from the notable bending of spine which was noted as the child was growing and episodes of per vaginal bleeding which were noted when the patient was 5 years old.

General examination revealed an otherwise healthy young girl, who was well oriented to her surroundings, with her spine bent to the right (scoliosis), irregular skin pigmentation on the right side of the chest and back, slightly enlarged breasts, vaginal bleeding and some sparsely distributed pubic hair. Her body stature, however, was normal for her age. On local examination of the craniofacial region, the patient had bilateral frontal bossing. She also had facial asymmetry due to a massive oval shaped exophytic mass on the right side of face that measured approximately 24 by 17 cm. The mass slightly crossed the midline, the overlying skin was hyperemic and shiny with visible dilated blood vessels. The right eye was displaced superiorly without any visual disturbance. The nose was displaced towards the left side, with occlusion of the right nostril. The overstretched overlying skin had normal temperature and could not be folded. The swelling was non-tender, bony hard and fixed to the underling structures (Fig. 1). Intraorally, the lesion was occupying the entire right side of the upper jaw extending just few millimeters beyond the midline to the left side of the palate. The lesion was oval in shape, with an otherwise normal overlying mucosa except on the anterior aspects where the mucosa was constantly dry due to exposure to external environment. The tumor caused displacement of all teeth on the right side of the upper jaw, however, these teeth were not mobile. Based on these clinical findings a provisional diagnosis of McCune-Albright syndrome was made.

**Fig. 2:**
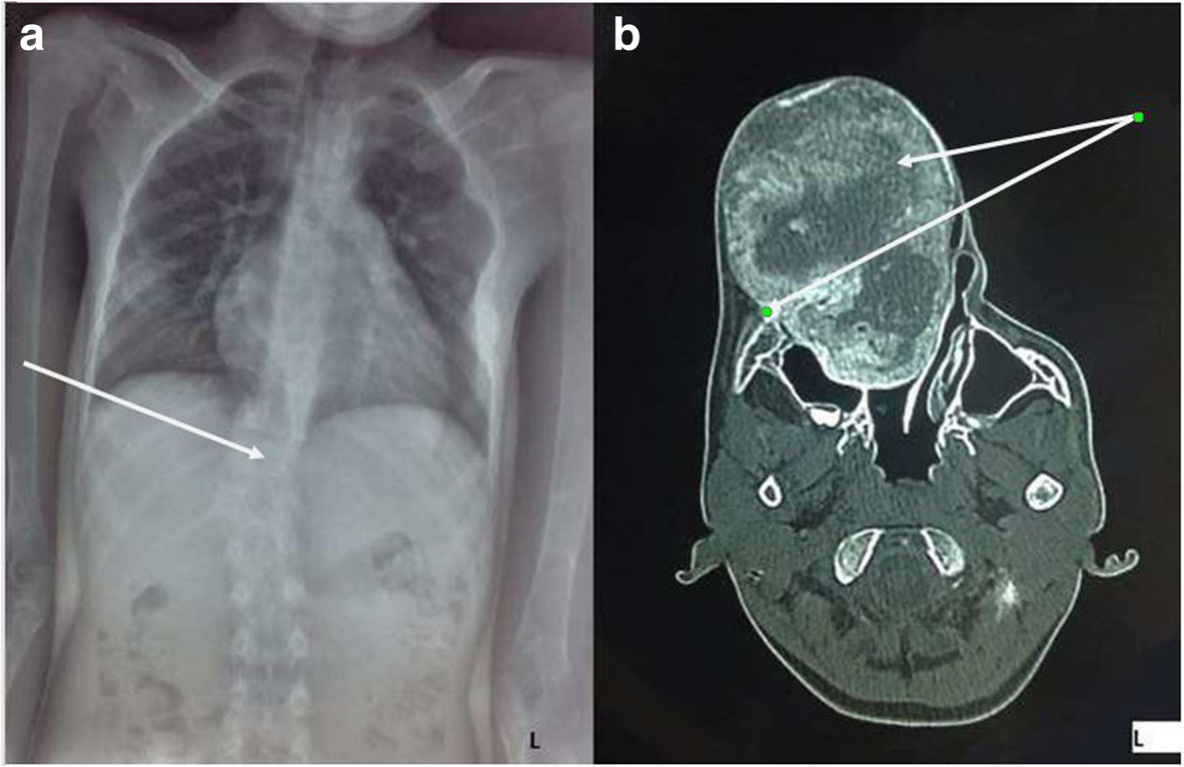
**a** Radiograph of the chest (PA view) of case 1 showing bending of the spine to the right (scoliosis). (see arrows) **b**: A CT-scan (axial cut) of case 1 displaying a mixed density lesion with areas of ground glass appearance (which was deep peripherally and light centrally) that involves the maxilla, palate and zygoma. The anteromedial wall of the right maxillary sinus is compressed laterally reducing the size of the maxillary sinus. The vomer is displaced laterally to the left, and the right nostril is completely obliterated

The work up done on the patient included histopathological analysis, skeletal survey (CT scan of craniofacial region and a skeletal survey of the body by conventional radiographs). Also complete blood count, calcium and phosphate levels in the blood, thyroid and parathyroid function tests and echocardiogram were done. The histopathological report of the specimen taken intraorally from the right maxilla revealed a moderately cellular fibrous stroma surrounding irregular curvilinear tubercle of woven bone arranged in Chinese letter pattern matching fibrous dysplasia. Skeletal survey with conventional radiographs showed scoliosis of the spine (Fig. 2a). The CT image displayed a mixed density lesion with areas of ground glass appearance (which was deep peripherally and light centrally) that involved the maxilla, palate and zygoma. The anteromedial wall of the right maxillary sinus was compressed laterally reducing the size of the maxillary sinus. The vomer was displaced laterally to the left, and the right nostril was completely obliterated (Fig. 2b). The complete blood count, calcium and phosphate levels and the thyroid function tests were all within the normal ranges.

**Fig. 3:**
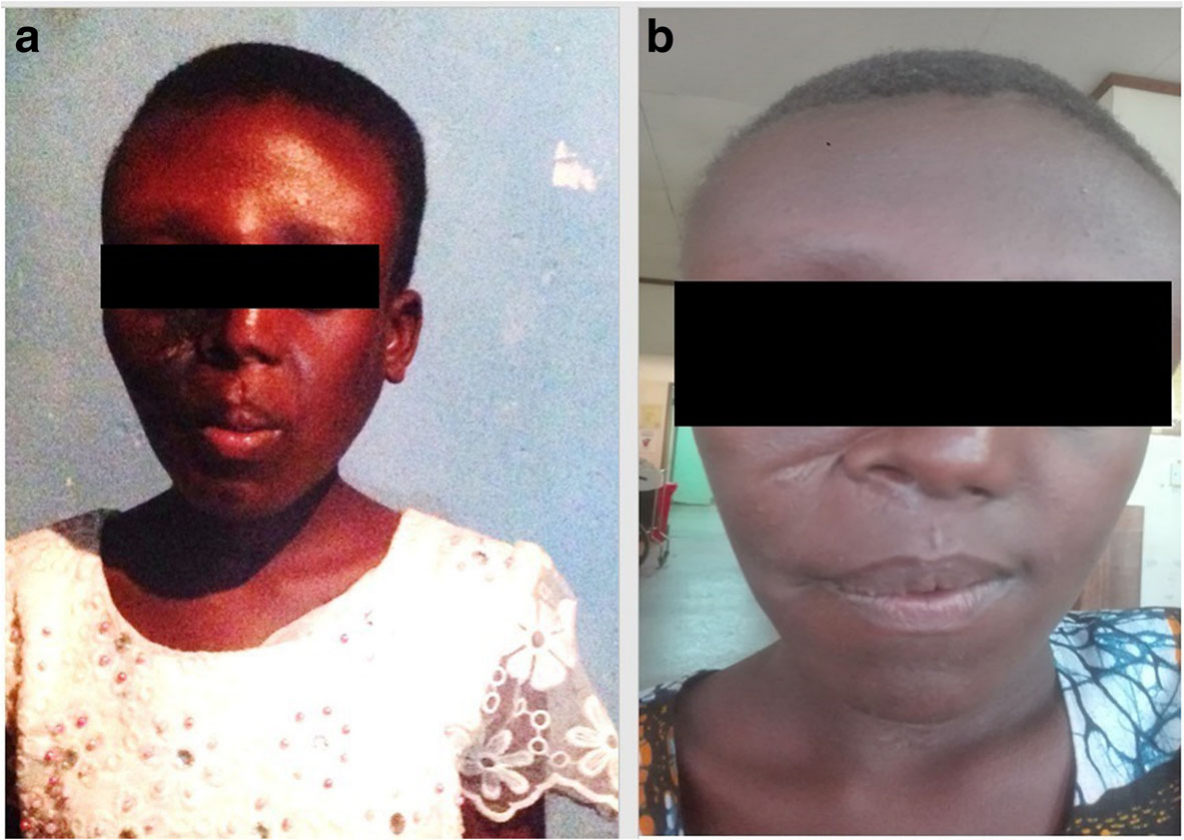
**a** & **b**: Case 1 two months (**a**) and two years (**b**) after surgery

The parents of the patient were counseled and treatment options were discussed including a series of corrective surgical procedures. Thus, upon consenting, in 2015, the first surgical procedure was carried out. Due to difficulty in intubation, tracheostomy was done and partial maxillectomy was carried out. The post-operative recovery was uneventful (Fig. 3a), and yearly follow ups were planned. In early 2017, the patient presented with a recurrent palatal swelling and a second surgery was done. The post-operative period was uneventful (Fig. 3b). Currently the patient is well and is under close follow up.

### Case 2

An 11-year-old male patient presented with complaint of a painless facial swelling on the right side of the face for about 4 years. The swelling started as a small hard mass in the oral cavity on the roof of the mouth. It was a painless swelling which gradually but progressively increased in size with resultant severe facial deformity that almost totally blocked the right nostril. Apart from the facial disfigurement, the patient twice experienced fractures of the lower right limb at 3 years intervals.

**Fig. 4:**
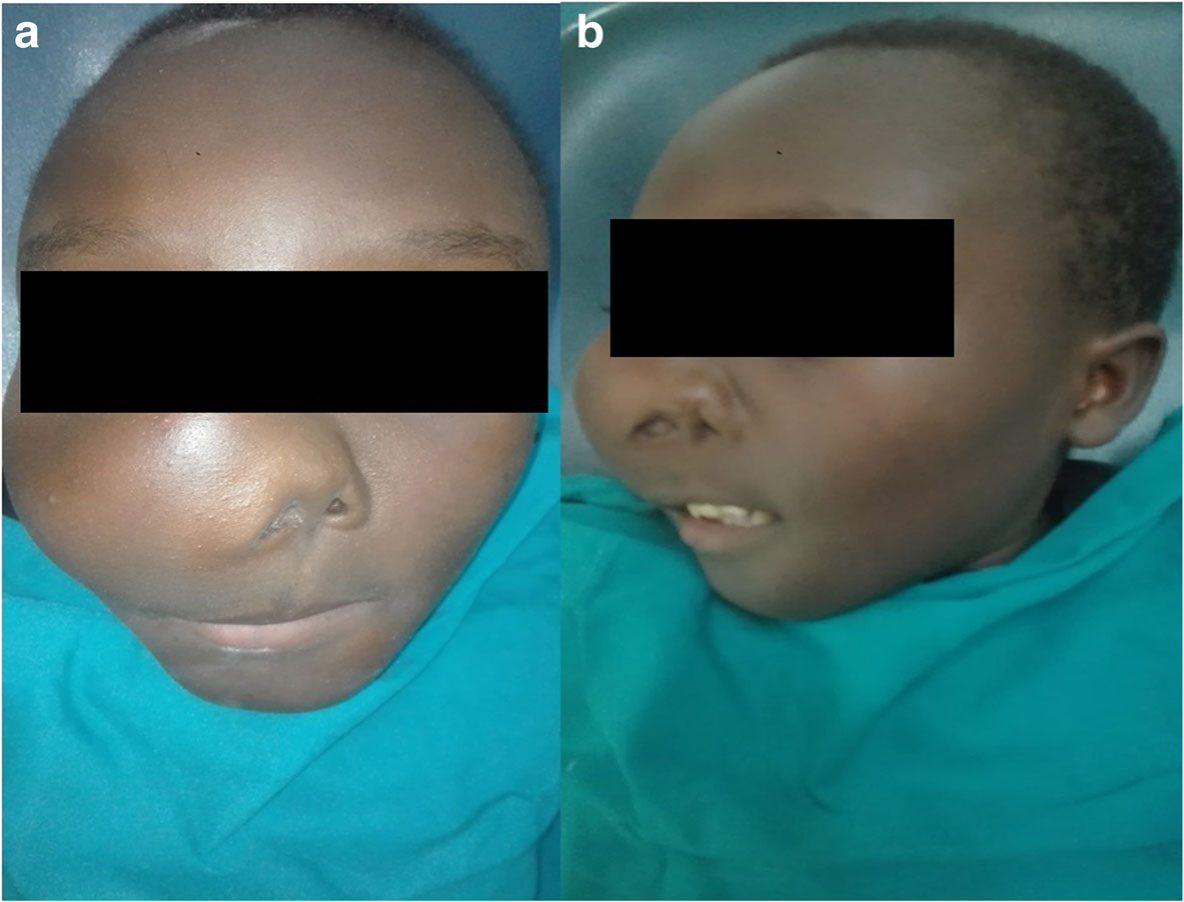
**a** & **b**: Case 2 showing extensive craniofacial lesion

Examination revealed a healthy young boy who was well oriented to his surroundings. He had a short stature with bowing of right arm and right leg that forced him to walk with the aid of clutches. Generally, he had normal looking skin with irregular skin pigmentation on the neck. On local examination of the craniofacial region, the patient had facial disfigurement due to a swelling on the midfacial region. There was an increase in inter-canthal distance with deviation of the nose to the left. The swelling had normal temperature, non-tender, bony hard and fixed to the underlying structures (Fig. 4). Intraorally, the lesion was on right side of the upper jaw, oval in shape, had normal overlying mucosa with more of palatal bone expansion compared to the buccal bone. The tumor caused some degree of displacement of teeth on the right side of the upper jaw without mobility. Based on these clinical findings a provisional diagnosis of McCune-Albright syndrome was made.

**Fig. 5:**
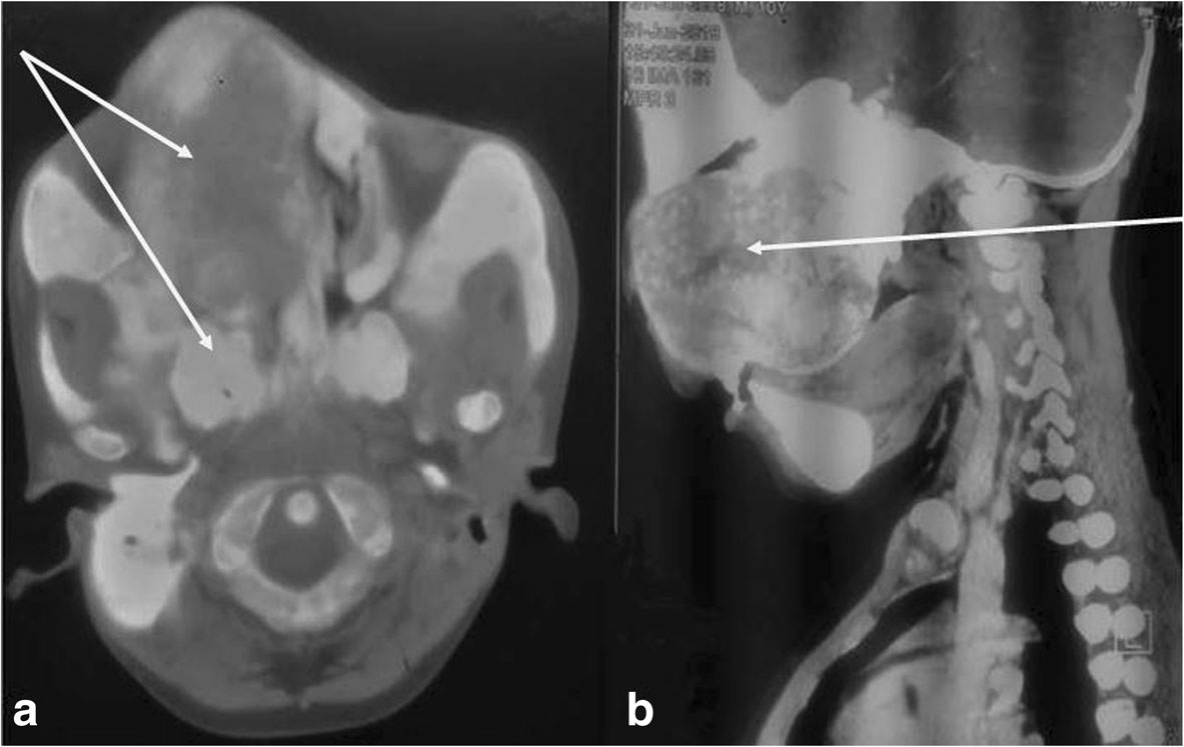
**a** & **b**: CT-scan (axial and sagittal cuts) showing a huge midfacial lesion which has a ground glass appearance involving the maxilla, palate, zygomatic bone, frontal bone and base of the skull. Both maxillary sinuses are obliterated. The lesion is filling the oral cavity

**Fig. 6:**
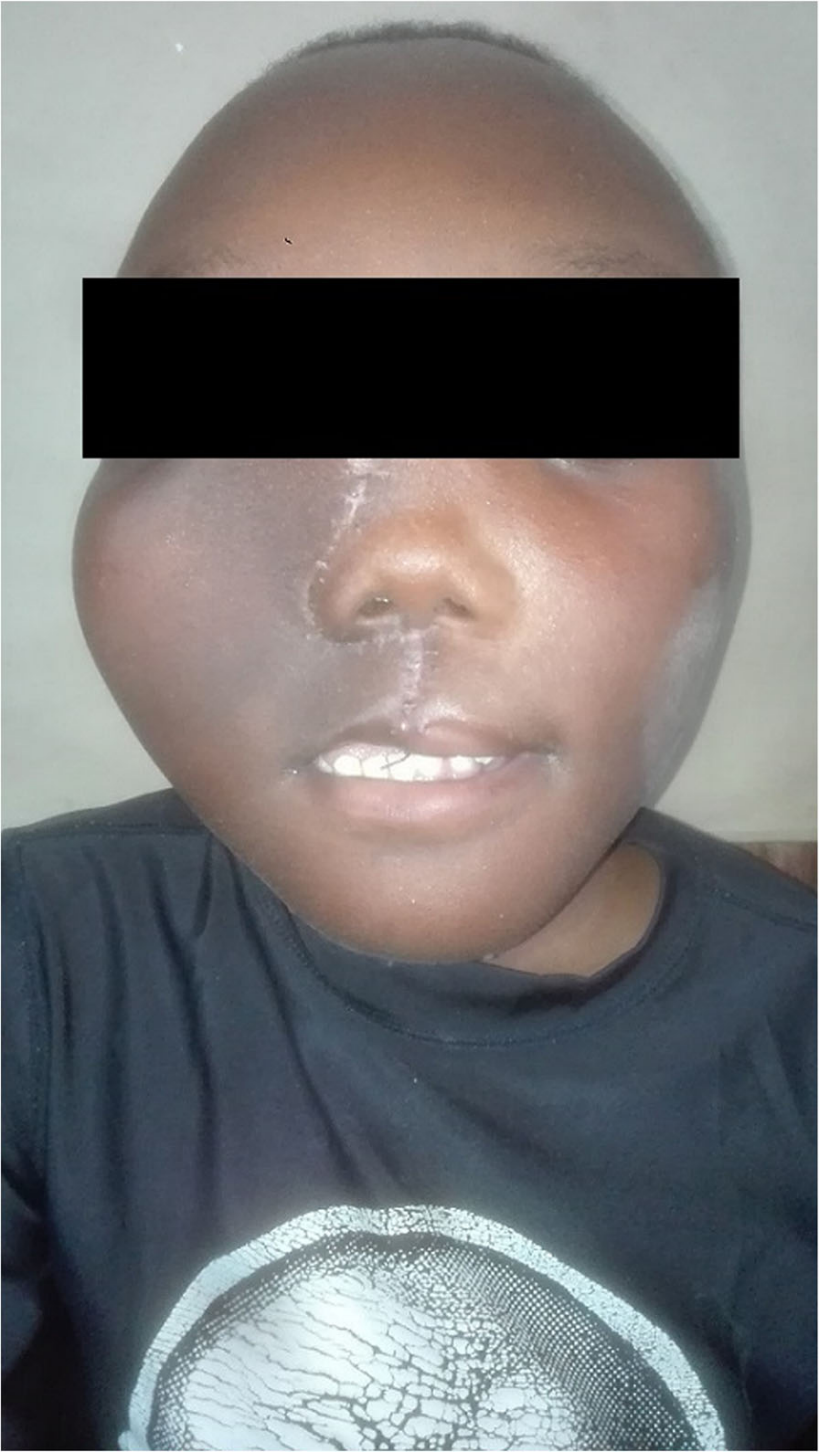
Case 2 one month after surgery

The work up done on the patient included, histopathological analysis, skeletal survey (CT scan of craniofacial region and conventional radiographs for rest of the body), complete blood count, calcium and phosphate levels in the blood, thyroid and parathyroid function tests, and echocardiogram. The histological features of the specimen taken intraorally from the anterior aspect of the maxilla were of moderate cellular fibrous stroma surrounding irregularly shaped bone trabecular with minimum osteoblastic rimming consistent with fibrous dysplasia. Skeletal survey showed bowing of upper and lower limbs. CT scan of head and neck region showed a ground glass appearing lesion that involved the maxilla, palate, zygomatic bone, frontal bone and base of the skull (Fig. 5a & b). Both maxillary sinuses were obliterated. The lesion was filling the oral cavity. The complete blood count, calcium and phosphate levels and the thyroid function tests were all within normal ranges. A series of surgical interventions to correct his disfigurement were planned. The first surgery (bone remodeling of the maxilla) was carried out in April 2018.The patient was discharged one-month post operatively (Fig. 6) and he is doing fine. The patient was advised to report back on 3 months basis for follow up.

## Discussion

Fibrous dysplasia is an idiopathic skeletal disorder in which medullar bone is replaced by poorly organized, structurally unsound fibro-osseous tissue [3]. Radiologically, FD is characterized by expansive lesions with endosteal scalloping, thin cortex, and an intramedullary tissue matrix showing a “ground class” appearance. FD lesions are typically not apparent at birth, but begin to manifest clinically during the first few years of life. The areas that most commonly display fibrous dysplasia are the proximal region of the femur and the skull base [10]. However, occurrence in the maxillofacial region is not uncommon. In the jaws it is more common in the upper jaw compared to the lower jaw. Occurrence in the upper jaw posses more challenges in the management and more morbidity to the patients given the complex anatomy of the midface. It is also well documented in the literature that FD can cause dental anomalies including tooth displacement and malocclusion with a prevalence of about 28% [11]. The two cases reported here presented with most of these features. Craniofacial swelling was apparent below 7 years of age in the two cases and both presented with malocclusion and skeletal deformities.

McCune Albright Syndrome was originally defined by the triad of polyostotic fibrous dysplasia of bone (FD), café-au-lait skin pigmentation, and precocious puberty and eventually endocrinopathies were added. Thus, this rare disease which is caused by postzygotic mutation of the GNAS1 gene, is considered as distinctive form of endocrine and non-endocrine neoplasia with affected cells being distributed in a mosaic pattern [1, 6]. Due to a unique molecular pathophysiology, there is inconsistency in the clinical features and severity of disease in different individuals [1, 8]. In the current reported cases, however, the patients had most of the classical symptoms without any endocrine manifestation.

It has been reported in the literature that in FD, pathognomonic lesion is appendicular skeleton fractures and bowing of long bones; in particular the proximal femur which may develop the classic “Shepherd’s crook” coxa-vara deformity, and moreover, scoliosis resulting from spinal FD commonly occurs [12, 13]. These findings were apparent in both the cases as one patient did present with scoliosis, while the other had history of multiple long bone fractures.

Although endocrinopathies have been reported as major components of MAS [1, 6, 10], in both cases reported here, thyroid and parathyroid hormone levels were within normal ranges, while precocious puberty was present in the first case. Precocious puberty (PP) is the most common endocrine manifestation of McCune-Albright syndrome being more common in girls than in boys [1], as evidenced in this report. Characteristically, the development of breast tissue is preceded by vaginal bleeding. In females, the elevated serum estradiol levels caused by intermittent autonomous activation of the ovaries is thought to be responsible for precocious puberty [6, 10]. In males, however, precocious androgen synthesis must be triggered by activating mutation in the Leydig cells, while testicular enlargement without signs of peripheral hyperandrogenism is caused by mutations of Sertoli cells only [1, 14, 15].

Café-au-lait skin macules presenting with irregular, sharp borders along the midline of the body may be apparent at or shortly after birth, and are considered as typically the first manifestation of MAS [12]. The café-au-lait skin macules are secondary to over production of melanin. Normally, pigment production in melanocytes is stimulated by melanocyte stimulating hormone acting through G_s_α/cAMP to induce expression of tyrosinase, the rate-limiting enzyme in melanin production. However, in MAS, melanocytes have increased levels of tyrosinase and numbers of dendrites and melanosomes due to activation of G_s_α/cAMP leading to overproduction of melanin [16].

Clinical studies on MAS are difficult because the condition is rare and clinically heterogeneous [17]. Genetic tests could aid in diagnosing MAF in patients with Café-au-lait skin macules at or shortly after birth to rule out the disease [4, 5]. Diagnosis challenges of MAS may be attributed to the pathophysiology of the disease, the somatic mutation in the gene GNAS 1 leads to G_s_α activation, hence elevated cAMP production. cAMP plays an essential role in various cell and body functions [18], thus differently affecting several systems of the body. Due to the fact that any organ of the body can be affected the work up of patients with MAF may necessitate several investigations, which may be an economic burden to the patients and their families, especially in developing countries. Moreover, lack of advanced molecular and genetic studies hinders diagnosis of the condition. However, due to cost and lack of expertise to conduct this kind of test is a setback in our setting as it is in most of developing world. In our cases plain x-rays were used to assess the status of the long bones and chest, while CT scans were useful in assessing the extension of the craniofacial FD, and its relation to the vital structures.

Treatment of patients with MAS entails a multidisciplinary approach, in which several specialties are required to work together. There is a great role to be played by Oral and maxillofacial surgeons, orthopedic surgeons, neurosurgeons, endocrinologist, cardiologists, dermatologists, neurologist, ophthalmologists, ENT surgeons and many others. The treatment of MAS in most cases is individualized rather than being specific, due to heterogeneity of the condition. The treatment option may be conservative management, medical and/or surgical management. In developing world, due to lack of specialists, these conditions go unnoticed in the initial phase. In conjunction with poverty and low knowledge on health-related issues in the communities, most patients present late when the condition is advanced, thereby making the treatment more complicated. Apart from above mentioned challenges, unavailability of certain types of medication recommended in treatment of the disease may render the management to be rather complicated.

In the cases reported herein, the patients presented about 4 years after the condition started and thus had huge facial tumors, which necessitated extensive and complicated surgeries.

## Conclusion

The reported cases illustrated the current clinical understanding of the consequences of disease activity and treatment of this remarkably diagnostically challenging disease in the developing world settings where both human and material resources are scarce. Delayed diagnosis and treatment of MAS result in a devastating physical disabilities and severe morbidity after treatment.

## Recommendations

Clinicians must put more efforts on measures towards early diagnosis, appropriate and adequate investigations for improved management of the disease and a close long term follow up. MAS requires a multidisciplinary approach so as to lessen complications and improve the quality of life of the patients.

## Data Availability

The complete data and materials described in the case report are freely available from the corresponding author on reasonable request.

## Abbreviations

ENT: Ear, Nose and Thorax
FD: Fibrous dysplasia
GH: Growth Hormone
MAS: McCune Albright Syndrome
